# Experiences of Family Members Supporting Adults With Type 1 Diabetes: A Qualitative Study

**DOI:** 10.1177/26350106251397924

**Published:** 2026-01-01

**Authors:** Peter Hellman, Anna Carin Aho, Charlotte Gillrell, Anne Wennick, Malin Axelsson

**Affiliations:** Faculty of Health and Society, Department of Care Science, Malmö University, Malmö, Sweden; Faculty of Health and Society, Department of Care Science, Malmö University, Malmö, Sweden; Lindeborg Primary Care Centre, Region Skåne, Malmö, Sweden; Faculty of Health and Society, Department of Care Science, Malmö University, Malmö, Sweden; Faculty of Health and Society, Department of Care Science, Malmö University, Malmö, Sweden

## Abstract

**Purpose:**

The purpose of the current study was to explore family members’ experiences of how to support an adult person with type 1 diabetes (T1D).

**Methods:**

A qualitative descriptive study with thematic analysis was conducted in Sweden between 2020 and 2021. Interview transcripts were analyzed from 13 people who are a family member (≥18 years) of an adult with T1D, and inductive coding identified salient themes. Family members were recruited via social media, and their length of time as a family member to an individual with the T1D ranged between 3 and 38 years (median 24).

**Results:**

The analysis and coding identified 4 salient themes: (1) searching for knowledge in unfamiliar situations, (2) applying new knowledge in everyday life, (3) balancing support to fit need and situation, and (4) over time, the support role matures.

**Conclusions:**

Families with members with T1D are complex, with relational and behavioral challenges arising as the members live their lives. Routines, habits, and unexpected events become especially challenging for families with a member with T1D. Because most self-care activities for adults are carried out within the family, directly involving family members in educational interventions is important. These interventions should include providing information about the symptoms and treatment for T1D, developing knowledge about how families can be affected, but also providing strategies for how families optimally implement necessary daily changes.

Type 1 diabetes (T1D) is a chronic disease that brings complicated challenges in everyday life. One challenge is to maintain an acceptable plasma glucose level to avoid acute life-threatening situations and future disease-related complications. Therefore, managing this disease involves not only the person with T1D but also members of the person’s family.^1^ The involvement of family members can make a difference by providing support and ensuring better self-management of the disease.^2^ However, for family members to contribute, they need to learn how to best support the person in managing the disease.

In a family, social roles, routines, and dynamics change when a member is affected with a long-term illness. Role modifications for both the person affected with the illness and their family may create tension in their relationship. The family can be viewed as a complex and interconnected system in which members interact with and mutually affect one another. This means that a disease such as T1D affects all members of a family.^3^ Family members often become involved and help plan daily activities based on the challenges posed by the illness. In many cases, the family’s routine needs to change, which may involve adopting a healthier and more regular diet, engaging in monitoring plasma glucose levels, and learning to recognize signs of impending hypoglycemia in the affected person.^4^ Hypoglycemia is described as a feared condition by adult persons with T1D^5,6^ and their family members.^7,8^ Therefore, it is important to learn how to be prepared to treat hypoglycaemia.^5,9^

How much interest different family members show in being involved in the management of T1D varies, and adults with T1D who experience a lack of support from family members can feel alone with the disease.^9^ Most T1D self-management takes place in the everyday life of the family; therefore, learning skills for prevention control and management are important for family members.^10,11^ Moreover, family members can provide a sense of stability and important support functions (ie, serve as a resource for the person’s diabetes self-management).^12^ However, the unmet needs of adult family members are reported to include the lack of educational interventions from health professionals with, among others, the means and strategies to learn how to support the person with T1D.^13^ Family members report experiencing a range of feelings, including worry, but they can also experience confidence and peace. In general, family members try to support the adult with T1D and not assume the role of a controller.^5^ This means that family members need to develop knowledge about how to balance their involvement in diabetes management and know when to step back to support the person’s autonomy in handling the disease.^5^ Studies report that in families with a member living with T1D, partners who become the “diabetes police” will result in negative effects. For example, studies show that poorer plasma glucose level control results when adult family members adopt a support role that involves criticism.^14^ Studies have also shown that adults with T1D adhere to regimes better and experience better quality of life when family members take a positive approach in their support.^15,16^ Although practical and emotional support from family members is usually appreciated by the adult person with T1D, the well-being of the family is also a concern. This means that the affected person may not always ask for help or talk about the disease in order to not worry family members.^9^

Research on families living with T1D often focus on families with children who have T1D during their early childhood and years up until adolescence or transition from adolescence to young adult.^17-19^ Research on family members of adult persons with T1D is scarce, with most studies in the field of diabetes focusing on families with children who have been affected by diabetes.^20^

The effects of support from adult family members are complex and can both help and hinder the management of T1D. Therefore, the support role of family members needs to better be understood in order to navigate the challenges that living with T1D entails.^13,21,22^ The current study’s focus on adults could potentially help health care providers develop effective strategies and educational interventions in situations involving families where an adult person is affected with T1D. Therefore, the purpose of the current study was to explore family members’ experiences of learning how to support an adult person with T1D.

## Method

### Study Design

A qualitative descriptive study design in accordance with Sandelowski^23^ was employed to create understanding of family members’ experiences of learning how to support an adult person with T1D. Data, collected through individual interviews, were analyzed with thematic analysis following Braun and Clark^24,25^ The consolidated criteria for reporting qualitative research (COREQ)^26^ checklist was used to ensure the integrity of the study design and data reporting. The current study was approved by the Ethical Review Authority (Reg. No. 2019-06330).

### Setting and Participants

The participants in the current study were recruited through adult persons diagnosed with T1D who were purposively sampled from social media sources to another study.^27^ The persons with T1D gave permission to the research team to contact their family members and invite them to the current study. In total, 13 family members of persons diagnosed with T1D were recruited to the current study. Inclusion criteria included adult (≥18 years) family members of a person with T1D who is able to understand and speak Swedish.

### Data Collection

Data collection was conducted from November 2020 until April 2021 through individual interviews. Persons diagnosed with T1D were asked to give written information about the study to family members and ask if they were interested in further information about the study. The contact information of those showing interest was then forwarded to 4 authors of the current study: PH, CG, MA, and AW. After having been informed verbally about the study and given the opportunity to ask questions, the family members were asked if they were interested in participating in the study. Those who gave their written informed consent were then booked for an individual interview at their convenience. The interviews were conducted digitally via Zoom (Version 6.2.50939; n = 1) or telephone (n = 12) by 4 of the authors. The participants could choose whether they wanted to be interviewed via Zoom or telephone. The interview via Zoom allowed the participant and the interviewer to see each other on the screen, but no observational data were collected because that was not the purpose.

All interviews started with an opening question asking the participant to narrate, from the perspective of being a family member, their experiences of how they as a family member had learned about T1D at the beginning and over time. The interviews lasted as long as the participants needed and ranged from 21 to 67 minutes (median 47 minutes); we used a semistructured interview guide (Table 1). They were audio recorded and transcribed verbatim in close connection with the interview, creating a total of 111 transcribed pages ranging from 5 to 12 pages (mean 8.5). Demographics were self-reported and are presented in Table 2.

**Table 1.** Interview Guide

Opening question	Can you tell how you learned about type 1 diabetes that your family member has?
Situation	Can you tell about a situation where you think it has been particularly difficult to be a family member to a person who has diabetes?
Probing questions related to situation	What did you think? How did you feel? What did you do in this situation? What experience did you bring with you from this situation? If you know what you know today—how would you have acted in the situation?
Time	Would you say that you have needed to change something in your life to be able to support your family member to manage her/his diabetes? If yes/if no, tell about it. The experience you have gained over the years/during the time you have been a family member to a person with type 1 diabetes—how have you used these experiences to be able to support this person to manage diabetes through life? You have told a lot about how you, based on your experiences, support your close relative to manage his/her diabetes. If you were to tell another family member to a person who recently has been diagnosed with T1D—what would you then tell that family member so she/he can support the person with T1D?
Probing questions to time	In the beginning? Later in life?
How to learn	Can you tell how you think you as a family member best have learned how you can support your close relative in managing his/her diabetes? What has made it easier/harder for you to learn how to support your close relative to manage diabetes through life?
Concluding question	Is there anything that you think is important to tell that we have not talked about?
Probing questions	How did you feel then? What were you thinking then? Can you describe? Can you give an example? Can you develop?

**Table 2.** Characteristics of Study Participants

Characteristics	No.
Participants in total	13
Female	8
Male	5
Participant’s role as family member	
Parent	6
Child	2
Sibling	1
Partner	4
Participant’s highest educational attainment	
College	2
University	11
Current employment status	
Employed	7
Student	2
Retired	4
Characteristics	Duration of type 1 diabetes diagnosis, y Median (range)
Duration of family member’s type 1 diabetes diagnosis	30 (10-45)
Duration of being a family member to an individual having the type 1 diabetes diagnosis	24 (3-38)

### Data Analysis

The analysis of the interview data was guided by thematic analysis, as described by Braun and Clarke.^24,25^ To enhance credibility, all members of the research team were actively engaged in the analytical process. This approach allowed for different perspectives to be considered during data interpretation, contributing to a more nuanced and trustworthy understanding of the findings. Regular discussions and joint reflections ensured consistency and transparency throughout the analysis.

**Figure 1.**
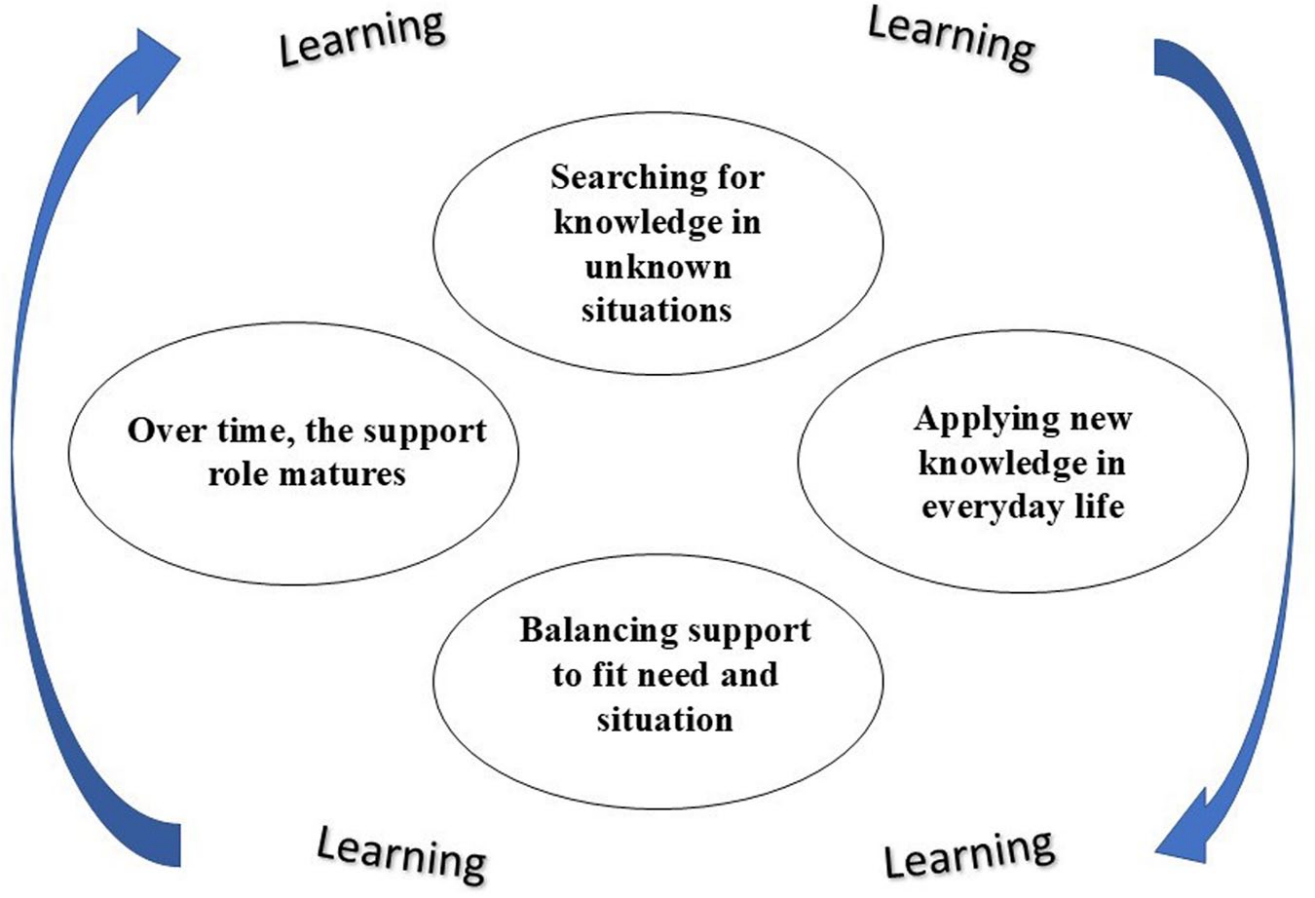
Central themes identified in the study regarding how family members support an adult person with type 1 diabetes, highlighting the continuous learning process involved in this support.

The interview transcripts were repeatedly read by the individual researchers to become acquainted with the content. During the reading, emerging thoughts and ideas were written down and then compared and discussed among the research team. Thereafter, the transcripts were methodically reviewed and content corresponding to the purpose was coded, followed by discussions for a richer and reflexive interpretation of the data. The codes were then organized into preliminary themes, the content of which was scrutinized and related to the data set in an iterative and collaborative process in the research team. The characteristics of each theme were carefully examined and further refined, leading to the final themes and their final positions in the thematic map illustrated in Figure 1, illuminating family members’ experiences of learning how to support an adult person with T1D. Examples of the analytical steps from data extracts to final themes are presented in Table 3.

**Table 3.** Data Extracts to Final Themes: Examples of the Analytical Steps From Data Extracts to Final Themes

Data Extract	Coding	Initial Theme	Final Theme
“The driving force was the desire to support the daughter as much as possible and to establish a sense of security, as greater knowledge about a subject increases confidence in managing it.” (Parent 12)	Knowledge fosters a sense of security and confidence	The new normal—in search of knowledge	Searching for knowledge in unfamiliar situations
“It is usually sufficient for him to consume a Dextrosol and a glass of milk for things to return almost to normal.” (Child 4)	The need to manage everyday life	Transition—managing balance in the new normal	Applying new knowledge in everyday life
“However, it must not be taken to excess. One must strive to maintain a balance so as not to overwhelm them—avoiding pressure or insistence that could lead to resistance or withdrawal.” (Parent 6)	The support provides a balanced form of compensation	Experiences being a closely related person	Balancing support to fit need and situation
“When you live in close proximity—when you share a home—you become acutely attuned to the other person. You know exactly how they move, how they speak, their tone of voice, expressions, and so on. You come to know a person inside and out.” (Partner 24)	Experience enables the early identification of warning signs	As time goes on, the supportive role matures	Over time, the support role matures

## Results

Four salient themes were generated: (1) searching for knowledge in unfamiliar situations, (2) applying new knowledge in everyday life, (3) balancing support to fit need and situation, and (4) over time, the support role matures.

### Theme 1: Searching for Knowledge in Unfamiliar Situations

At the beginning of their relationship, regardless of family ties, none of the partners had enough information about T1D. All the family members described feelings of uncertainty regarding how to act in situations where knowledge about T1D was necessary and having to constantly search for information whenever in T1D-related situations. This became the new normal (ie, when a family member was diagnosed with T1D or when a family member became aware that a family member suffered from T1D, they all expressed a need for knowledge). Some family members explained that the need was driven by a sense of helplessness and others by a sense of responsibility regarding the person’s well-being (ie, wanting the best for the person with T1D). Family members also experienced the need to create a sense of security, in the sense that the information they gathered would lead to knowledge about how to handle T1D-related situations in everyday life.

If you don’t have knowledge about what you’re supposed to take care of, you can experience insecurity and uncertainty, which makes everyday life difficult. The more knowledge you have, the more secure you become in daily life. (Parent 12)

Family members gathered information about T1D from different sources. For some family members, health care professionals in hospital settings constituted a source and context of information in connection with the diagnosis when information was given orally and written. Other family members added that the family member affected by T1D constituted the main source of information and that this information was mostly given orally. However, the family members explained that they felt a commitment to also seek out other sources of information on T1D research news (ie, new methods of treatment and development of different types of insulin), for example, literature with a focus on diabetes, such as books, journals, and newspapers.

No, but since he is . . . of course, I try to perk up my ears if something happens regarding diabetes, maybe listen a bit more attentively, now some new research comes out, they believe this and that, then I perk up my ears. (Parent 11)

Peer support in the form of the sharing of information and experiences with other family members in similar situations via diabetes associations was also described.

The Diabetes Association was also a resource, but perhaps not so much for expertise, but as support and a sounding board. (Parent 5)

However, according to some family members, online forum discussions on diabetes associations’ websites that were unsupervised by health care personnel were considered not to be a source of reliable information that could be applied in everyday life.

### Theme 2: Applying New Knowledge in Everyday Life

As everyday life continued, the family members started to incorporate information and newly gained knowledge from experienced T1D situations into day-to-day living, which reflects that the family members had become more comfortable with T1D in everyday life. Applying the knowledge they had gained enabled family members to integrate T1D self-management, thus handling and balancing the risk for hypoglycemia with everyday activities. As family members described the need to integrate T1D with everyday life to support the person, they experienced that planning for day-to-day living needed to be carefully integrated with everyday activities, sports, and in particular, situations in which other illnesses affected the person with T1D. Hypoglycemia is dangerous and potentially life-threatening, which means that the worry of fluctuations in plasma glucose levels were always present when planning activities and thus often monitored. The families developed strategies that built on experiences of how to avoid such fluctuations, generating a gradual capability to manage the family member’s plasma glucose fluctuations in everyday life. The family members gave various examples of how they planned activities.

It’s the planning to bring all the equipment for the pump and insulin, so it becomes an extra packing, like, to make sure you have everything, and it needs to be stored in. (Partner 10)

Another example of leisure activities where previous experiences played a role in the planning was, for example, traveling. Family members argued that they contributed to the planning for any situation that could happen to the person with T1D during travel. They gave various examples of how they proactively planned (eg, contacting hospitals in advance as a precaution and packing double doses of insulin in separate suitcases).

No, we have traveled a lot . . . so we made sure that, before we arrived, we established contact with our friend’s doctor so that we had a quick way into health care if something were to happen, for example. (Parent 12)

In certain situations, T1D significantly affected daily life, which led to family members needing particularly good planning. This was especially the case during times when the person with T1D became afflicted with fever or stomach flu and had difficulties maintaining self-management.

Yes, but you have to try to pull yourself together and make sure she gets her insulin, stays hydrated, and gets what she needs, like a sugar solution if she gets a stomach bug and things like that. (Parent 39)

In such cases, the family members needed to be ready to use their knowledge about how to balance food intake, insulin dosage, and blood sugar testing.

### Theme 3: Balancing Support to Fit Need and Situation

According to family members, although the person with T1D could mostly manage T1D-related situations by themselves, when they needed support, adequate and, importantly, balanced support was needed. Family members experienced that they needed to learn how to balance support and appropriately attune it to the situation; otherwise, they risked overcompensating. Balancing support to fit need and situation required that family members learned from experience how to compensate in a timely and adequate way. The family members explained that it was important to learn how to balance their supportive roles in each situation. If the support was more active than needed, then it could trigger irritation and risk conflict with the person with T1D. Therefore, family members explained that it was important to strive for balanced support.

No. I mean, being supportive is essential, you know, you have to . . . you have to be involved all the time, and you can’t be uninterested or nonchalant and such, but you really have to show that you care, I think. But it shouldn’t go overboard so that it becomes . . . it shouldn’t be too much for them, but you have to try to balance it so that you don’t push and pressure and so on . . . so that it doesn’t backfire. (Parent 6)

Maintaining and not disrupting established routines, such as eating healthily, exercising, and getting enough sleep, were mentioned by family members as important for the person with T1D and as such, a way to manage and prepare for daily situations. Therefore, adapting their support and not affecting these routines was considered important.

She has to make the decisions . . . when we meet, I also try to think about the routines . . . mostly so that I don’t mess anything up for her, so I try to support what she already has, to go along with it, or to have days that work well according to her routines, so I might adapt a bit to the way she already lives. (Sibling 40)

Being the family member of a person with a potential deadly disease had generated experiences through situations where the person affected by T1D was not able to manage the situation themselves. In such situations, the family members had learned how to become supportive in such a way that they actively avoided taking part in any situation that may escalate and become life-threatening. In such situations where this support was needed, it was regarded as vital support.

But she herself maybe couldn’t think clearly all the time, so I tried to find out and tell her to go here and there and do this and that, and remind her to check her blood sugar all the time and monitor her urine and all that. (Parent 39)

There were experiences when the family member, due to conditions such as pregnancy, took on a more active supportive role than before. The family member had a more passive role up until the pregnancy.

Since my partner has had diabetes her whole life, she became my expert in a way, so if I have any questions, I asked her. But I went with her . . . when she was pregnant, I went with her to a dietitian, and I talked . . . I also attended some doctor’s appointments where doctors talked about how it affects a diabetic who becomes pregnant. (Partner 7)

Being a support also involved learning to manage the nutritional aspects of T1D, which included adherence to a strict intake of carbohydrates not only for the person with T1D but also for their family members. The family members explained that because they had to balance the support long term (ie, also be subjected to strict dietary restrictions that benefit those with T1D), this had negative consequences on their own well-being. These became evident when the family members experienced difficulties switching to a more normal and flexible intake of carbohydrates later in life.

### Theme 4: Over Time, the Support Role Matures

The family members’ experiences of T1D-related situations in daily life increased over time, which meant that their supportive role was continuously tested and thus could develop. Family members could then trust in experiences that had led to the development of knowledge and skills needed to support the self-management of the person with T1D. Being a family member means that T1D was a challenge for the whole family, which had a profound effect on their daily life. Even though the disease had manifested itself differently among the families and caused varying degrees of symptoms, one aspect was common: The impact of the disease eventually reached a level where it could be fully integrated into family life. Family members expressed this differently, but the essence is captured in the following quote:

I think the most important thing to say is that type 1 diabetes is a disease that you can learn to live with and live a very good life. I believe that. Life is not over. (Parent 13)

Experiencing and mastering multiple T1D-related situations in daily life meant that the supportive role of the family member could enter a more mature stage—one that involved the skills to think and act in an overarching proactive fashion. The family members had learned to observe and interpret specific and subtle signs indicating, for example, a fluctuation in plasma glucose levels.

As a relative, you need to learn the signals and know what to do. It’s very important. You can’t go to the hospital for everything; you need to know what to do. (Parent 11)

Then it becomes . . . I know when you live closely together, if you live together, you know exactly how the other person is . . . their body language, how they talk, what they say, what tone they use, expressions, and so on. You know the person inside and out. (Partner 24)

Being the family member of a person with T1D meant being involved in the support of the self-management of diabetes in many ways and having been faced with many T1D-related situations and concerns. With experience, the participants in the study learned skills that made them capable of managing and solving T1D-related situations in everyday life. Based on previous experiences, over time, and in some cases, over a lifetime, solution-oriented and stepwise ways to handle and solve situations developed.

So a solution-oriented approach but also breaking it down into smaller steps, so to speak. And I would say that reduces my anxiety because it makes the situation feel a bit easier to handle. (Child 4)

This development, which helped reduce anxiety among family members, had gradually strengthened the support role of the family, and an inherent feeling of being able to live life as any other family would result.

## Discussion

The results of the current study show that when a person became a member of a family where an adult has T1D or learned that an individual in the family had been diagnosed with T1D, a period followed where the family member needed to seek information related to and about T1D. The family members applied this information in daily life situations to support the affected person’s T1D self-care. The family members’ T1D-related experiences and the information they had acquired were used to provide T1D self-care support. This support was important to maintain at the right level, balancing it with the needs of the person with T1D. Based on their T1D-related experiences, an insight among the family members was that with their support, T1D could over time be incorporated into the family’s daily life.

The purpose of the current study was to highlight family members’ experiences of learning to support an adult with T1D. Considering the family members’ perspective is important because most of the self-care and disease management occurs within the family domain. The individuals in the current study were all members of a family in which an adult had been affected by a chronic illness, namely, T1D. Managing a chronic illness will pose challenges in a family for both the family members and the affected adult.^16^ Family members shared experiences of their efforts to understand both the disease and its impact on the affected person and how they, as family members, learned to support the affected person’s self-care of the disease and integrate it into the daily life of the family. In some cases, as shown in the current study, they actively compensated for the family member’s inability to perform self-care, such as during a drop in plasma glucose levels. The participants interviewed in the current study all expressed, to varying degrees, that they were part of the self-care of T1D that their family member was affected by. This aligns with other studies that also show that family members actively support and influence self-care.^7,28^ Family members learn to influence the person’s self-care in many ways, such as by generally improving well-being, sharing responsibility, and providing instrumental support, for example, by helping with insulin injections.^29^ In conversations and discussions within a family, attitudes and opinions are conveyed, and family members often have a significant influence on the person’s psychological well-being and their willingness and ability to follow recommendations, such as medical advice, and to maintain exercise and a healthy diet.^29,30^ This influence can also have a negative impact, and the participants in the current study reported how they needed to balance the support given; otherwise, it could have negative consequences. Support that involves a large, overly controlling, and overprotective element (eg, criticizing food choices) has been shown to be related to both high levels of diabetes-related stress and poorer dietary habits in the affected person.^31^ Given that families are complex social and emotional entities, family interventions should invite and include all members, with a focus on how educational interventions for T1D can aid in the functioning of the family and its support role.^29,32^

It is important to reduce the stress that arises in a family due to necessary changes in the family’s daily life routines.^33^ In line with this finding, family members in this study reported that the family’s daily life needed to undergo changes to integrate the disease. The family members in the current study all had experiences of feeling stressed by the affected person’s diabetes. Managing a chronic illness like T1D is challenging not only for the person with the disease but also for members of their families. The emotional and physical demands of managing T1D can create anxiety in family members when they are in situations where their knowledge of diabetes is insufficient.^34,35^ Not only is knowledge about the disease itself necessary but also knowledge about how a chronic illness will profoundly affect the family’s daily life long-term. Educational interventions explaining why changes in daily life are necessary can help manage emotional aspects that often arise in daily life when family members discover that change is needed.^36^ Moreover, it is important that the interventions aimed at family members also include tools on how to optimally change family routines.^13,37^

The participants in the current study expressed that the managing of T1D eventually became possible to fully integrate into the family’s daily life. It is reasonable to assume that family members can experience such full integration when their learned support skills have been tested and fine-tuned repeatedly in connection with T1D-related situations and they gain the insight that their supportive skills are sufficient for daily life to be lived despite T1D in the family. Based on our results, educational interventions showing how the support role of family members can contribute to integrating the T1D disease into family life seems therefore relevant to develop. However, studies involving family members or focusing on the integration of T1D within the family are sparse. The focus tends to be more on the affected person, with only occasional reports of outcome measures from family members.^13^

### Methodological Strengths and Limitations

To strengthen the trustworthiness of the study in accordance with Lincoln and Guba,^38^ a series of measures were implemented. With the aim of being transparent about the conditions within the research group and providing insight into the selection method and how the data were analyzed, COREQ^26^ was used. Although the sample size in the study was small, there were variations in the population’s experiences of learning to support an adult with T1D as a family member in terms of both experiences from different types of relationships (as a parent, partner, child, or sibling) and the duration of these relationships (Table 2). Data collection was conducted via telephone (n = 12) and Zoom (n = 1). When interviews are conducted using the telephone or a digital tool like Zoom, this can result in the loss of nonverbal data, such as body language. However, it seems unlikely that nonverbal data would have affected the results of the current study, particularly because the use of the telephone as a data collection method has been shown to lead participants to share more information than they would have in a face-to-face interview.^39^ A study by Ward et al^40^ reported that telephone interviews in qualitative data collection methods are fully reliable to use as a data collection method. In connection with the current study, a high degree of reliability was sought through the use of a common interview guide by the members of the research group who conducted the interviews, all of whom have previous experience conducting interviews in qualitative studies. All members of the research group are registered nurses: One is specialized in diabetes care, one is specialized in pediatric care, and the others have experience working as nurses in various contexts. Furthermore, all interviews were recorded electronically to capture all details and thereby reduce the risk of nuances being lost. As accounted for, the participants in the current study were sampled from persons with T1D who were recruited via social media, which on one hand, may limit the transferability of the findings due to self-selection bias but on the other hand, may enhance transferability by reflecting a broader and more diverse population.

## Conclusion

Families with members with T1D are complex, with relational and behavioral challenges arising as the family and its members live their lives. The routines, habits, and unexpected events of life exist in all families but become especially challenging for families where a member is affected by T1D. Most self-care activities for adults are carried out within the family and therefore involve family members. Therefore, it is important to directly involve family members in educational interventions. Educational interventions should include information about T1D, its symptoms, and its treatment principles. However, they should also develop knowledge about how a family, being a complex system, should implement necessary changes in daily life.
